# Comparison of the effects of Crataegus oxyacantha extract, aerobic exercise and their combination on the serum levels of ICAM-1 and E-Selectin in patients with stable angina pectoris

**DOI:** 10.1186/s40199-015-0137-2

**Published:** 2015-12-19

**Authors:** Leila Jalaly, Gholamreza Sharifi, Mohammad Faramarzi, Alireza Nematollahi, Mahmoud Rafieian-kopaei, Masoud Amiri, Fariborz Moattar

**Affiliations:** Department of Exercise Physiology, Islamic Azad University of Khorasgan, Isfahan, Iran; Associate Professor in Exercise Physiology, University of Shahrekord, Shahrekord, Iran; Subspecialist of Cardiology and Assistant Professor, Shahrekord Univercity of Medical Sciences, Shahrekord, Iran; Medical Plants Research Center, Shahrekord Univercity of Medical Sciences, Shahrekord, Iran; Health Research Center, Shahrekord Univercity of Medical Sciences, Shahrekord, Iran; School of Pharmacy and Pharmaceutical Sciences, Isfahan University of Medical Sciences and Health Services, Isfahan, Iran

**Keywords:** Aerobic exercise, Cratagol, ICM-1, E-selectin, Stable angina pectoris

## Abstract

**Background:**

Adhesion molecules play an important role in the development and progression of coronary atherosclerosis. The aim of this study was comparing the effect of Cratagol herbal tablet, aerobic exercise and their combination on the serum levels of Intercellular adhesion molecule (ICAM)-1 and E-Selectin in patients with stable angina pectoris.

**Methods:**

Eighty stable angina pectoris patients aged between 45 and 65 years, were randomly divided into four groups including three experimental groups and one control group: aerobic exercise (E), Crataegus oxyacantha extract (S), aerobic exercise and Crataegus oxyacantha extract (S+E), and control (C). Blood sampling was taken 24 h before and after 12 weeks of aerobic exercise and Crataegus oxyacantha extract consumption. The results of serum levels of ICAM-1 and E-selectin were compared.

**Results:**

Intergroup comparison of the data revealed a significant reduction (*P* <0.01) in serum levels of ICAM-1 and E-selectin in experimental groups. Analysis of data showed that the serum levels of ICAM-1 had significant difference when group S+E was compared with groups S and C, but not group E (*P* = 0.021, *P* = 0.000 and *P* = 0.068, respectively). Also the difference between the levels of E-selectin was significant comparing S+E and S but not E with group C (*P* = 0.021, *P* = 0.000 and *P* = 0.052, respectively).

**Conclusions:**

Twelve weeks effects of aerobic exercise and Crataegus oxyacantha extract consuming is an effective complementary strategy to significantly lower the risk of atherosclerosis and heart problems.

**Electronic supplementary material:**

The online version of this article (doi:10.1186/s40199-015-0137-2) contains supplementary material, which is available to authorized users.

## Background

Stable angina pectoris is a type of ischemic heart disease described by the discomfort (pain rarely called) in the depth of the chest which has no specific location [1]. The heart coronary inflammation and atherosclerosis are the main causes of chest angina [2, 3]. As mediating agents during different stages of the atherosclerosis, adhesion molecules play important roles in leukocyte recruitment to the surface of vascular endothelium especially in the earlier stages of atherosclerosis. In addition, in atherosclerosis region the leukocyte recruitment on the endothelial cell surface is performed by Cell adhesion molecules (CAMs). As a result, inflammatory agents and adhesion molecules can cause insufficient blood circulation in myocardial tissue and the lack of oxygen leads to the disintegration of these tissues resulting in unstable improper performance of biochemical, mechanical and electrical activities of myocardium. Thus eliminating or reducing of these agents can increase the availability of oxygen to tissues and may help to reduce the heart problems. Among the adhesion cells that have been proved their role in cardiovascular disease especially in atherosclerosis, are Intercellular adhesion molecule (ICAM)-1, Vascular cell adhesion molecule (VCAM)-1 and E-selectin [4, 5]. Intercellular adhesion molecules (ICAMs) are cell surface glycoproteins that in inflammatory condition expressed on a wide variety of cell types including leukocytes, epithelial cells, endothelial cells and fibroblasts while, they are low-expressed in the normal conditions in the vascular endothelial cells, lymphocytes, and monocyte [6].

The demonstration of soluble E-selectin in blood can thus be considered as conclusive evidence of endothelial activation. E-selectin facilitates the earlier stages of polymorphonuclear adhesion to the endothelial cell, constituting an early serum marker of the inflammatory response and promoting cellular damage by ischemia [7]. In the prevention and treatment decade, more attention has been focused on the role of inflammatory factors in the development of atherosclerosis [8]. Therefore the chemical drugs such as non-steroidal anti-inflammatory drugs (NSAIDs) have been used frequently for management of this condition. The meta-analysis of these non-steroidal anti-inflammatory drugs has showed their strong relation with the risk of myocardial infarction, shock and heart stroke [9]. Among the common anti-inflammatory drugs herbal medicines are well known. Several botanicals including Crataegus oxyacantha extract has been shown to play a role in the improve of cardiovascular diseases such as hypertension, hyperlipidemia, and in particular, congestive heart failure [10–13]. In this regard, it was found that these effects may in part be due to the presence of antioxidant flavonoid components [1, 12]. According to conducted researches in attempt to discover new ways to reduce these diseases, it seems that Crataegus oxyacantha extract can be effective to reduce inflammatory adhesion molecules such as ICAM-1 and E-selectin that are new indicators in the development and progression of cardiovascular disease.

Aerobic training can caused reduction in serum levels of ICAM-1 and E-selectin, too [14]. However, the changes in serum levels of CAM-1 and E-selectin in response to aerobic exercise reported conflictingly in various research findings [15–18]. Nevertheless, as far as we know, there is no report on the effect of Crataegus on plasma inflammatory factor and adhesion molecule levels. Walker et al. and Yuen et al. [19, 20] studied the effect of aerobic exercise in interaction with plant Crataegus, but in these studies only the favorable effects on blood pressure, asthma, and enhanced exercise capacity after consumption of herbal Crataegus were reported.

The probable effect of herbal remedy in combination with physical activity is the method, which can be considered to be special. Because of the importance of this issue, this study was aimed to focus on anti-inflammatory effects of Crataegus oxyacantha extract and aerobic training in patients with stable angina to determine how efficient are these methods, and to focus on the effect of Crataegus oxyacantha extract for the first time in combination with aerobic exercise on serum ICAM-1 and E-selectin.

## Methods

### Crataegus oxyacantha extract

Patients were given the Crataegus oxyacantha extract in Cratagol tablets form. Cratagol was produced at Goldaru pharmaceutical company (Isfahan, Iran) as a Coated tablets in packs of 30 tablets containing 240 mg of dried extract of Crataegus (Hawthorn) leaves and flowers that had been standardized as 4–6 mg Vitexin-2- ramnozide per each tablet. Crataegus oxyacantha extract in Cratagol tablets was produced at EPO Istituto Farmochimico Fltoterapico S.r.l. company (20141 Milano, Italy) (Additional file 1: Appendix A. Supplementary Information). Also Cratagol tablets containing adjuvants Avicel, corn starch, talc, magnesium stearate and Arvzyl. In addition, the researchers had no organizational or financial dependence to the company manufacturing crataegus extract.

### Placebo tablets

Placebo tablets were prepared from Goldaru pharmaceutical company (Isfahan, Iran). The placebo was prepared from granulated of inert powder of lactose and the core of pressed tablets was coated similar to that of Cratagol and the produced tablets were entirely similar to the original product in terms of color, size and appearance. The tablets were encoded and they were used in clinical studies through the Double Blind Method. Original and placebo tablets were previously coded and after the required experiments the type of drugs based on their codes in studied subjects were determined, then statistical analysis were performed.

### Expriments

**Fig. 1:**
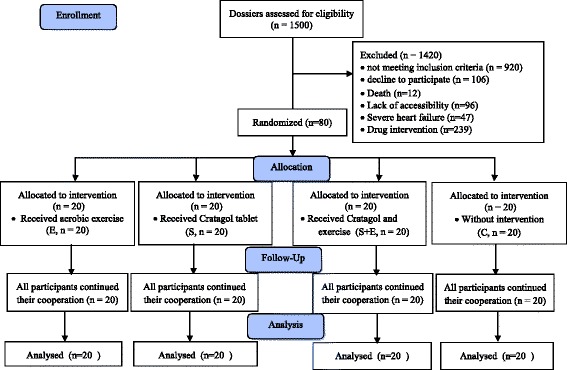
Flow diagram of the trial

Subjects were included patients with stable angina who were under treatment at the Imam Ali cardioligy sub-specialty polyclinic of Shahrekord, Iran. The diagnosis of stable angina pectoris in the patients was performed by Professor of Cardiology with coronary angiography test. Patients whose angiography test history was carried out between 1 and 12 months ago and there were no new symptoms of pain or discomfort during this period and also their coronary atherosclerosis was less than 50 %, with clear treatment history within past 3 months, aged between 45 and 65 years were selected for this study. 1500 potential cases of stable angina patients who were under treatment at the Imam Ali cardioligy sub-specialty polyclinic of Shahrekord, Iran were investigated. Most of these patients were out of our entrance requirements, also unwillingness to participate the study, severe heart failure, lack of accessibility, pharmacological interventions and death were of the criteria for which the participants were eliminated and also patients with severe heart failure and/or treated with digoxin, cisapride, anticoagulant, anti-arrhythmic drugs were excluded. Finally, 80 patients (44 male and 36 female) suffering from ischemic heart disease of stable angina were recruited and randomly assigned into four groups (20 patients (11 male and 9 female)): three experimental groups including aerobic exercise and placebo (E), Crataegus oxyacantha extract (S), aerobic exercise and Crataegus oxyacantha extract (S + E) and one control group (C) and were followed for 12 weeks. Patients in each group were homogenate with regards to age, sex, weight, and Body Mass Index (BMI). Because of the relationship between individual characteristics such as sex, age, BMI, and weight and inflammatory markers measured in this study, we attract the reviewer’s attention to the fact that the researchers tried to reduce bias in the results by choosing four homogenated groups. In particular, in order to excluding the effect of sex on the results, the number of males and females were equal in both groups, however, the distribution of men and women based on their number was accidental. Random number tables were used where each value was randomly selected with an equal chance of choosing any integer among 1–44 (men) and 45–80 (women) by QuickCalcs online calculator (http://www.graphpad.com/quickcalcs/randomn2.cfm) (Additional file 2: Table S1 and S2), which allocated the numbers randomly to control and experimental groups. Also, the amount of physical activity, nutrition, diet, smoking, alcohol, symptoms and duration of angina were accurately determined through medical record questionnaires and reports contained in patient records. As well as, we had no crossover or contamination between groups and the current study was carried out as multiple parallel groups until the end of experiments. The patients in all 4 groups were administered with Methoral (50 mg/day), Aspirin (80 mg/day), and sublingual Nitroglycerin in case of discomfort. Crataegus oxyacantha extract were additionally taken by patients in the experimental groups (S) and (S+E), twice a day, and each time one tablet along with chemical medication with a little water before meals. The control group (C) did not take any Crataegus oxyacantha extract. The control group in this study was not influenced by any intervention and also did not receive the placebo tablets, but during the experimental period, the weekly report of their nutritional status and physical activity were recorded and were under control. This project was approved by the ethics committee of the Medical University of Shahrekord, Iran and was recorded in the clinical trial center with registration number IRCT201303098435N1. The participants had sufficient information and awareness to participate in the study and the process by which they filled out the consent form was controlled and documented (Fig. 1).

### Exercise program

Aerobic exercise program was performed on a treadmill (after one week pre-adaptation) with an intensity of 40–60 % of heart rate reserve (HRR) and a rating of perceived exertion (RPE) of 11–13 (on 6–20 Borg scale), twice a week, each time 20–30 min, for 3 month (12 week) [21]. Meanwhile, The patient’s conditions and emotions, intensity and duration of exercise, blood pressure, resting and exercise heart rate were controlled at the beginning, during, and after exercise by digital stethoscope (Geratherm Medical AG, German) and recorded. The exercise was stopped in case of discomfort in the chest, asthma, dizziness, fatigue and loss of systolic blood pressure more than 10 mm Hg and the exercises were continued, modified or interrupted under the opinion of psychiatrists. The data obtained from exercise and blood tests were collectively recorded in separate sheets for final analysis. Training exercise was carried out by specialized expert, under the direction of Professor of Cardiology in the Imam Ali sub-specialty polyclinic of Shahrekord, Iran.

### Blood tests

Fasting blood test was performed 24 h before study and also after 12 weeks. Blood tests were performed in specialized laboratories from 5 ml blood sample of arm vein of each patient. After 5 min of coagulation time, the samples were centrifuged at 3000 rpm for 10 min. Immediately blood tests were performed to determine the levels of ICAM-1 and E-Selectin in serum using a detection kit in specialized laboratories.

### Measurements

Serum concentrations of ICAM-1, and E-selectin were carried out using standard ELISA Kit (Boster, USA), with a sensitivity of 10 pg/ml and 4 pg/ml respectively. An ELISA reader (Ststfax 2100, USA) was also used.

### Statistical analysis

Data were analyzed using SPSS 17 and the results presented as mean ± standard deviation. Comparisons between groups were made using the Kolmogorov-Smirnov test as well as ANOVA followed by Least significant difference (LSD) test. A two-sided *P*-value of 0.05 was considered as statistically significant.

## Results

The data were classified according to average and standard deviation in intervention and control groups. The Kolmogorov-Smirnov test showed normal distribution of data in all stages of pre-test and post-test. Statical analysis showed that there were not any significant baseline difference in serum levels of ICAM-1 between groups (F = 0.28, *P* = 0.834). Also, there were not any significant baseline difference in the levels of E-selectin between groups (F = 0.97, *P* = 0.411).

The result of descriptive data for inflammatory factors mean difference in each group, as well as the result of t- test related to control and experimental groups between pre-test and post-test are presented in Table 1. A significant decline (*p* <0.01) was observed in the mean difference between pre-test and post-test in all three experimental groups including aerobic exercise (E), Crataegus oxyacantha extract (S), combination of aerobic exercise and consumption of Crataegus oxyacantha extract (S+E) in the levels of ICAM-1 and E-selectin, while in the control group the decrease due to intake of chemical drugs did not show any significant difference (ICAM-1, *P* = 0.412 , E-selectin, *P* = 0.313) (Table 1).

**Table 1:** Descriptive data t-test between pre-test and post-tes

Variables	Group(*n* = 20)	Means ± SD		*P*
		Pre-test	Post-test	
ICAM-1(ng/ml)	E	65.5 ± 39.7	20.8 ± 2.7	0.001
	S	61.3 ± 38.1	23.1 ± 3.7	0.001
	S+E	90 ± 53.5	21.9 ± 3.9	0.001
	C	56.1 ± 36	42.1 ± 33.1	0.412
E-selectin(ng/ml)	E	3.2 ± 1.5	1.8 ± 1	0.001
	S	3.4 ± 1.9	2.2 ± 1.3	0.003
	S+E	3.5 ± 1.3	1.8 ± 0.7	0.001
	C	2.7 ± 1.3	2.4 ± 1.2	0.313

Aerobic exercise ((E), *n* = 20, 11 males, 9 female), Crataegus oxyacantha extract ((S), *n* = 20, 11 males, 9 female), Aerobic exercise and Crataegus oxyacantha extract supplements ((S + E), *n* = 20, 11 males, 9 female), Control ((C), *n* = 20, 11 males, 9 female), significant (*P* <0.05) in all groups except control (C)

Statical Analysis of data showed a significant difference in serum levels of ICAM-1 in experimental groups (F = 5.91, *P* = 0.002). Post hoc test showed that the serum levels of ICAM-1 had significant difference when group S + E was compared with groups S and C, but not group E (*P* = 0.021, *P* = 0.000 and *P* = 0.068, respectively). Also, significant differences in the levels of E-selectin was observed among experimental groups (F = 3.34, *P* = 0.023). Post hoc test showed that the difference between the levels of E-selectin was significant comparing S+E and S but not E with group C (*P* = 0.021, *P* = 0.000 and *P* = 0.052, respectively).

According to the weekly reports of patients’ condition during the study (12 weeks) there was not seen any side effects and evaluation of this case is out of our duty.

## Discussion

The purpose of this study was comparing the effect of Crataegus oxyacantha extract consumption, aerobic exercise or their combination on the serum levels of ICAM-1 and E-Selectin in patients with stable angina pectoris.

One of the important finding of the present study was the reduction in serum levels of ICAM-1 and E-Selectin due to aerobic exercise. The changes in serum levels of ICAM-1 and E-selectin in response to aerobic exercise reported conflictingly in various research findings but Studies have shown that ICAM-1 and E-selectin have a similar relationship [15–17, 22–24]. Much evidence suggests that inflammation plays important role in the process of atherosclerosis [16]. During inflammation process, cytokines such as TNF-a, IL-1B, IL-6, IL-10, IL-1ra, sTNF-R are produced and activated at the site of inflammation [8]. In the adhesion step, when leukocytes are approaching the side of endothelial cells, molecules such as VCAM- and ICAM-1 [25] and E-selectin [26] accumulated. Changes in adhesion molecules such as ICAM-1 and E-Selectin are closely related to leukocyte accumulation and inflammatory factors C-reactive protein (CRP), Interleukin (IL)-6, Tumor necrosis factor (TNF)-a, and Creatine kinase (CK). So the activity of endothelial injuring markers is influenced by inflammatory factors [15]. The increased expression of ICAM-1 is dependent on nuclear factor-kappa B (NF-KB) And the increased level of cytokines particularly Interleukin-1 beta (IL-1B), is responsible for the production of E-selectin [6, 7, 27]. Studies showed that physical activity can reduce the resting levels of these cytokines by reducing obesity, leptin and increasing the adiponectin and insulin sensitivity [16, 28–30]. Exercise training can effect on adhesion molecules and endothelial injuring markers by decreasing of inflammatory factors particularly NF-KB and IL-1B [22, 31].

One of the most important finding of this study was the reduction in the serum levels of ICAM-1 and E-selectin after taking Crataegus oxyacantha extract. Crataegus extract via its protective effect against oxidative stress caused by released free radicals, which in turn it can improve cardiac function and reduce infarct size in a rat model of prolonged coronary ischemia and reperfusion [10]. Chen et al. [32] stated that Crataegus improved endothelial function so exposed anti hypertension properties. Crataegus stabilizes collagen function trough prevention of its degradation by secreted enzymes from leukocytes during inflammation, so the adverse effect of atherosclerotic plaques and produced endothelial injury markers on vessels can be prevented. it also inhibit the action of free radicals so improves coronary artery dilation and blood supply to the heart muscle via prevention the activity of phosphodiesterase-Cyclic adenosine mono-phosphate enzyme resulting in increase of cyclic Adenosine monophosphate (AMP) in myocardium and increase of its contractile strength. It increases the time of excitability of heart muscle by blocking potassium channels, which ultimately will lead to improved arrhythmia [33, 34].

Of the other important finding in this study was the reduction in serum levels of ICAM-1 and E-Selectin due to aerobic exercise along with Crataegus oxyacantha extract consumption. Walker et al. [19] were examined the promising hypotensive effect of Crataegus extracts. In this study, the blood pressure values at rest, after exercise and after a stress test were evaluated which both of systolic and diastolic blood pressure decreased in all experimental groups. In addition, the desire to reduce anxiety in those who had received Crataegus was seen compared to the other groups [19]. Crataegus extract in comparison with placebo increased the exercise tolerance in the congestive heart failure. Since the patients during the treatment period were not exposed to any other cardio-active drug, it was proved that advancing in exercise tolerance was due to the action of the Crataegus extract [20]. Exercise program improves environmental inflammatory markers associated with endothelial dysfunction such as soluble intercellular adhesion molecules and vascular, colony factor stimulating of granulocyte and macrophage and chemo attractant protein 1 by increasing of available nitro oxide [16, 27, 35]. Because of its anti-hypertension property, Crataegus can help to improve endothelial function and to reduce the production of inflammatory markers and adhesion molecules through inducing endothelium-dependent no-mediated vasorelaxation by eNOS phosphorylation [12, 32, 36]. Physical activity decreased the oxidative stress by modifying the anti-oxidative defenses in serum [37–39]. In addition, the prescription of Crataegus extract through its protective effect against oxidative stress can result in improvement of cardiac function [10, 40, 41]. Endurance activities and reduction in carbohydrate storage of the body increase the epinephrine, norepinephrine, the growth hormone, and cortisol by the endocrine system, resulting in increase of lipid oxidation [42], While the other part of the beneficial effects of Crataegus can be considered for reduction of lipid peroxidation and oxidative stress in vascular tissue [10, 40, 43]. Since adipose tissue is one of the main locations of secretion the inflammatory markers and cytokines, endurance exercise in combination with Crataegus consumption increases lipolysis rate and decreases the body fat, so it can be considered as an effective strategy to reduce inflammatory mediators and cell adhesion molecules [11, 44]. This may explain the observed stronger effect in the combination group (S+E). Therefore in this study, the exercise along with consumption of Crataegus oxyacantha extract resulted in double reducing of plasma ICAM-1 and E-selectin. While we could not fully control diet, rest, exercise and mental stress outside the clinic environment.

## Conclusion

Our findings suggest that aerobic exercise and Crataegus oxyacantha extract over a period of 12 weeks significantly reducing of plasma Cell adhesion molecules in patients with stable angina pectoris. However, since aerobic exercise and herbal drug induces many physiological processes in the body, evaluation of the interaction between aerobic exercise and herbal drug effects in patients with heart disease needs further study.

## Additional files

Additional file 1:
**Appendix A.** (DOCX 6257 kb) Additional file 2:
**Appendix B. Table S1.** Random numbers between 1 and 44 (males). **Table S2.** Random numbers between 45 and 80 (female). (DOCX 23 kb)
