# Supplementary material for: Comparison of the effects of Crataegus oxyacantha extract, aerobic exercise and their combination on the serum levels of ICAM-1 and E-Selectin in patients with stable angina pectoris
Source: Daru. 2015 Dec 19;23:54. doi: 10.1186/s40199-015-0137-2 (PMC4684934; doi:10.1186/s40199-015-0137-2)
Supplement: Additional file 1: — Appendix A. (DOCX 6257 kb) [file 40199_2015_137_MOESM1_ESM.docx]

Appendix A.
