# Supplementary material for: Comparison of the effects of Crataegus oxyacantha extract, aerobic exercise and their combination on the serum levels of ICAM-1 and E-Selectin in patients with stable angina pectoris
Source: Daru. 2015 Dec 19;23:54. doi: 10.1186/s40199-015-0137-2 (PMC4684934; doi:10.1186/s40199-015-0137-2)
Supplement: Additional file 2: — Appendix B. Table S1. Random numbers between 1 and 44 (males). Table S2. Random numbers between 45 and 80 (female). (DOCX 23 kb) [file 40199_2015_137_MOESM2_ESM.docx]

Appendix B.

**Table S1.** Random numbers between 1 and 44 (males)

| **Row #** | **A** | **B** | **C** | **D** |
| --- | --- | --- | --- | --- |
| **1** | 28 | 44 | 12 | 3 |
| **2** | 15 | 26 | 38 | 42 |
| **3** | 40 | 39 | 32 | 24 |
| **4** | 7 | 41 | 16 | 30 |
| **5** | 37 | 13 | 9 | 35 |
| **6** | 19 | 1 | 31 | 8 |
| **7** | 4 | 25 | 11 | 22 |
| **8** | 29 | 10 | 5 | 17 |
| **9** | 2 | 14 | 33 | 6 |
| **10** | 20 | 34 | 18 | 23 |
| **11** | 36 | 21 | 27 | 43 |

Each value was randomly selected, with an equal chance of choosing any integer between 1 and 44 (males). Column A: Aerobic exercise ((E), n=20, 11 males, 9 female), Column B: Crataegus oxyacantha extract ((S), n=20, 11 males, 9 female), Column C: Aerobic exercise and Crataegus oxyacantha extract supplements ((S+E), n=20, 11 males, 9 female), Column D: Control ((C), n=20, 11 males, 9 female).

**Table S2.** Random numbers between 45 and 80 (female)

| **Row #** | **A** | **B** | **C** | **D** |
| --- | --- | --- | --- | --- |
| **1** | 48 | 76 | 59 | 60 |
| **2** | 67 | 70 | 73 | 68 |
| **3** | 80 | 53 | 50 | 63 |
| **4** | 58 | 51 | 55 | 49 |
| **5** | 61 | 66 | 56 | 78 |
| **6** | 64 | 77 | 69 | 57 |
| **7** | 46 | 72 | 52 | 62 |
| **8** | 79 | 74 | 65 | 54 |
| **9** | 75 | 71 | 47 | 45 |

Each value was randomly selected, with an equal chance of choosing any integer between 45 and 80 (female). Column A: Aerobic exercise ((E), n=20, 11 males, 9 female), Column B: Crataegus oxyacantha extract ((S), n=20, 11 males, 9 female), Column C: Aerobic exercise and Crataegus oxyacantha extract supplements ((S+E), n=20, 11 males, 9 female), Column D: Control ((C), n=20, 11 males, 9 female).
